# Sanitary Condition and Longevity of Jews

**Published:** 1882-02

**Authors:** 


### Selections.

### SANITARY CONDITION AND LONGEVITY OF JEWS.

We believe that we have been able to show that nearly everywhere the Jews enjoy the following physiological immunities, when compared with the inhabitants of the countries in which they are established: 1. Their general fecundity (the relation of births to population) is less. 2. According to locality, their marriages are more or less fertile, but everywhere they preserve a larger number of their children. 3. They have much fewer illegitimate births and infants born dead. 4. The proportion of male births is sensibly higher. 5. Their mortality is less, and their mean life is longer. 6. Their increase, by the preponderance of births over deaths, is more rapid. 7. If they do not completely escape contagious diseases, they are less severely affected by them. 8. They are protected from certain diseases, such as pulmonary phthisis and scrofula. They become acclimatized and reproductive under every latitude.

These immunities resist the generally miserable condition which all observers attribute to them, as they do the frequency of their consanguineous marriages and their sojourn in cities, remote from the salutary influences of rural life enjoyed by the majority of the other inhabitants. What can be the causes of these privileges? Must we regard them, with a large number of authors. as the result of a superior vitality inherent to the race, which has been preserved intact through ages, and in spite of the difference of climates, in consequence of an almost complete absence of crossing? Must we seek these only in the persevering observation of the laws of hygiene laid down in Deuteronomy? But these rules were only applicable to the climate under which the ancient Israelites lived, whether in Judea, or in Egypt during the captivity. Or should we attribute them, first, to the salutary influence of marriage, which Jews contract at a less advanced age than Christians, and also to their following the less fatiguing occupations, and which are but little exposed to accidents? Can we admit, also, that they profit by the beneficial hygienic effects the spirit of order and economy, of regularity in habits and moderation in tastes, in the relative severity of their manners and the completely private and family life which many observers attribute to them? Finally, are we to admit, with others, that their state of misery is only in appearance, and that, in reality, their well-being is generally superior to that of the population amidst which they live? All these hypotheses are admissible.

On the other hand Dr. S. Gibbon, medical officer of health for the Holborn district, London, in his report for the last year, says, that whatever may be the cause, there is no doubt that the Jew's life in London is, on the average, worth twice as many years as a Christian's. The Hebrews of the metropolis are notoriously exempt from tubercular and scrofula taint. It is very rare that one meets with pulmonary consumption among them. The medical officer of one of their large schools has remarked that their children do not die in anything like the ratio as Gentile children; and in the district of White-chapel the medical officer of health has reported that on the north side of High street, occupied by the Jews, the average death-rate is twenty per thousand, while on the south side, occupied by English and Irish Gentiles, it is forty-three per thousand.—*Revue Scientifique.*

